# Near-Infrared Carbon Nanotube Tracking Reveals the Nanoscale Extracellular Space around Synapses

**DOI:** 10.1021/acs.nanolett.1c04259

**Published:** 2022-08-29

**Authors:** Chiara Paviolo, Joana S. Ferreira, Antony Lee, Daniel Hunter, Ivo Calaresu, Somen Nandi, Laurent Groc, Laurent Cognet

**Affiliations:** † Université de Bordeaux, Institut d’Optique & Centre National de la Recherche Scientifique, UMR 5298, 33400 Talence, France; ‡ Université de Bordeaux, Interdisciplinary Institute for Neuroscience, UMR 5297, 33076 Bordeaux, France

**Keywords:** Single-walled carbon nanotubes, brain extracellular space, single particle tracking, superlocalization microscopy, organotypic brain slices, synapses, correlative imaging

## Abstract

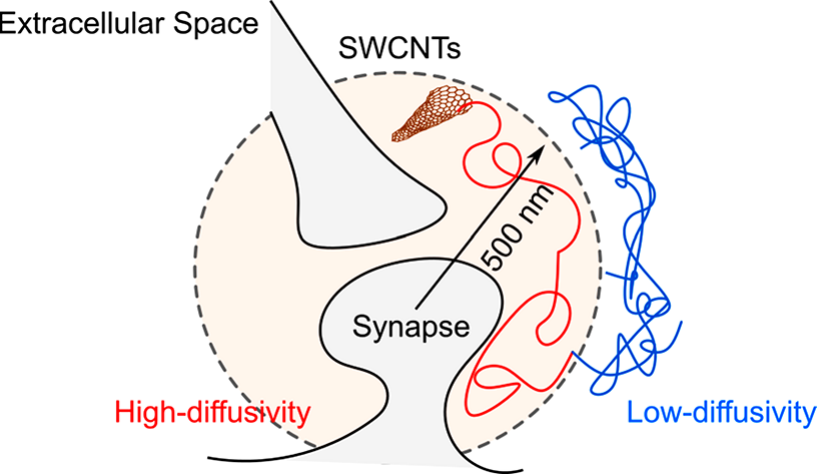

We provide evidence of a local synaptic nanoenvironment in the brain extracellular space (ECS) lying within 500 nm of postsynaptic densities. To reveal this brain compartment, we developed a correlative imaging approach dedicated to thick brain tissue based on single-particle tracking of individual fluorescent single wall carbon nanotubes (SWCNTs) in living samples and on speckle-based HiLo microscopy of synaptic labels. We show that the extracellular space around synapses bears specific properties in terms of morphology at the nanoscale and inner diffusivity. We finally show that the ECS juxta-synaptic region changes its diffusion parameters in response to neuronal activity, indicating that this nanoenvironment might play a role in the regulation of brain activity.

Neuronal communication in the central nervous system mainly occurs at the level of synapses through the release of neurotransmitters in the synaptic cleft and the activation of postsynaptic receptors. Neurotransmitters can spill over from synapses and act at a distance through a process known as “volume transmission”, in which signaling molecules navigate within the brain extracellular space (ECS).^1,2^ Despite technical and molecular advances over the past decades, the local dimensions and architecture of this complex environment have yet to be elucidated in identified regions of the living brain. In particular, the design and combination of experimental strategies offering nanoscale resolution and sectioning capabilities are needed to correlate the narrow and tortuous environment with specific cellular structures.^3^

Changes in the ECS can affect neuronal excitability and signal transmission by altering local ion concentrations in the healthy and diseased brain.^4−6^ Compared with free diffusion in an “open space” where molecules move randomly, diffusion in the ECS is critically dependent on the physical and chemical structure of the local microenvironment.^7^ Zheng et al. investigated the extracellular diffusivity inside the cleft of synapses formed by hippocampal mossy fibers which could be resolved by diffraction limited microscopy approaches, suggesting reduced diffusion when compared to free medium.^8^ Yet, understanding the mechanisms through which the ECS modulates neuronal communication, particularly around excitatory synapses at the nanoscale, still represents a key challenge in brain research.^3^ Because the characteristics of the ECS around synapses remain unknown at the submicron scale in native live brain tissue, it is important to establish whether the synaptic ECS environment displays specific dimensions and diffusional properties.

To tackle this question, we developed a correlative imaging approach based on single-particle tracking of individual fluorescent single wall carbon nanotubes (SWCNTs) in living brain tissues and on speckle-based microscopy of synaptic labels (Figure 1A). The first approach provides information about ECS dimension and diffusion properties at the nanoscale, while the second one allows us to identify and localize postsynaptic densities inside brain tissues. Using this method, we revealed the existence of a local synaptic nanoenvironment (which we called “juxta-synaptic” region) lying within 500 nm of the postsynaptic densities (PSDs). This local ECS environment is defined by specific properties in terms of morphology at the nanoscale and inner diffusivity. We finally show that the juxta-synaptic region changes its diffusion parameters in response to neuronal activity, indicating that its features might play a role in the regulation of brain activity.

**Figure 1:**
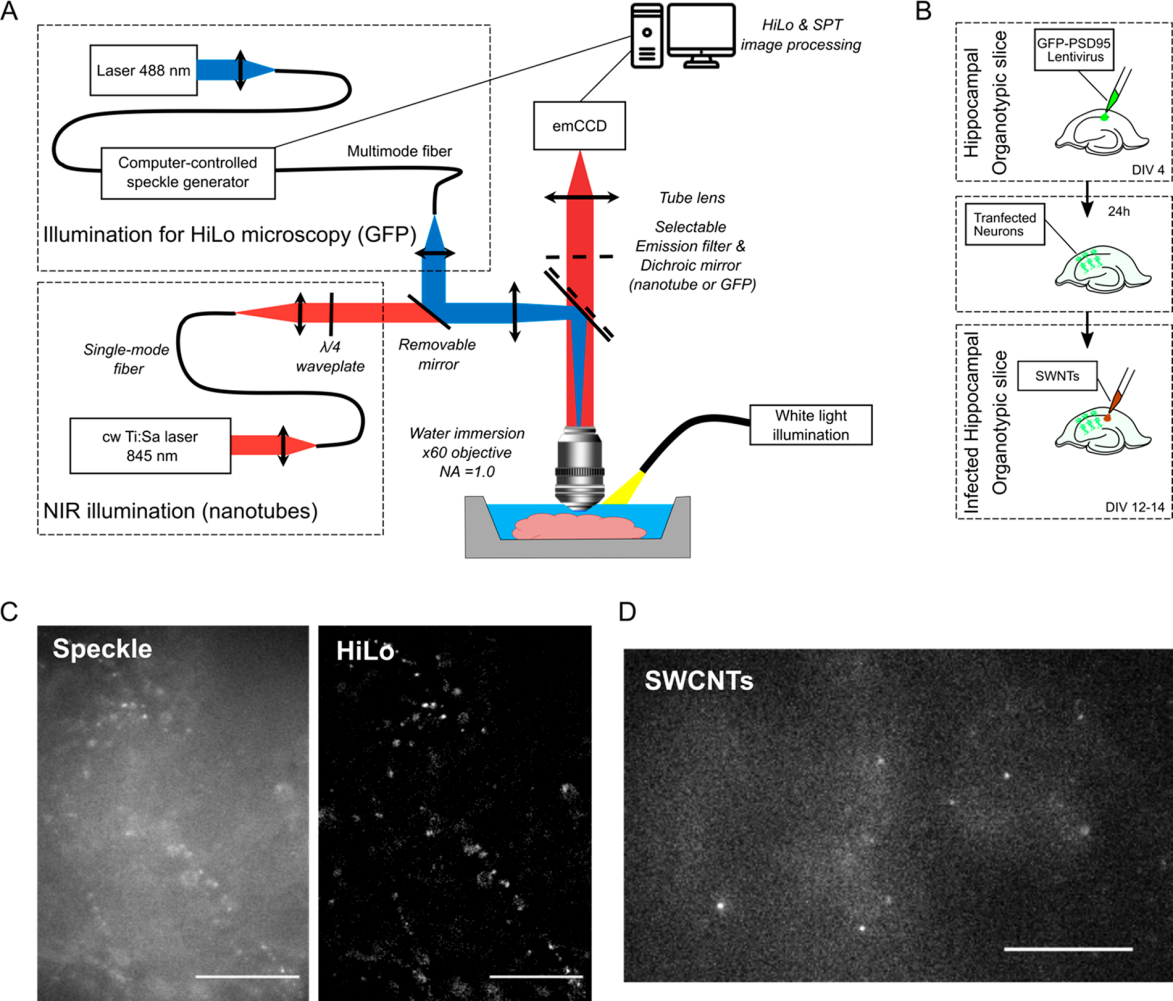
Experimental conditions. (A) Schematic of the experimental setup. Imaging was performed on a customized epifluorescent microscope equipped with a speckle based illumination setup. NIR imaging of SWCNTs was performed at 845 nm with a 60× immersion objective. GFP-PSD95 expressing neurons were imaged at 488 nm through the same objective and recorded by a HiLo modality. Individual SWCNTs were followed in the ECS of living brains using an EM-CCD camera. A standard white light fiber and low magnification objective (4×) were initially used to check the CA1 position in the hippocampal slice. (B) Schematic of the organotypic slices. Rat organotypic cultures were prepared from rat pup brains and cultured for 4 days *in vitro* (DIV) before PSD95 infection. SWCNTs were incubated at DIV 12–14 for 2 h. (C) Example of a speckle and HiLo images recorded with the Sparq system. Individual GFP-PSD95 clusters were clearly recognizable. Scale bars are 20 μm. (D) Typical recording of SWCNTs in living organotypic slices. Nanotubes were sparsely and individually detected at high signal-to-noise ratio. Scale bar is 20 μm.

Near-infrared luminescent SWCNTs are interesting nanoparticles for deep brain tissue microscopy,^9^ due to their unique brightness, photostability, and near-infrared emission range in the biological window.^10^ SWCNTs also demonstrated the capability to locally probe *in situ* chemical species, including neurotransmitters.^11^ At the single nanotube level, we have recently shown that SWCNTs can access ECS in the brain tissue of young^12^ and adult^13^ rodents, and their diffusion trajectories can reveal ECS local dimensions at the nanoscale. It is noteworthy that another optical approach based on STED microscopy^14^ could provide similar structural ECS dimensions. Using these optical methodologies, ECS remodeling in a neuropathological condition was successfully reported.^14,15^ Furthermore, contrary to other microscopy techniques displaying nanoscale resolution for the study of structural tissue features (also including electron microscopy), SWCNT tracking allows us to investigate ECS architectures in intact living brain at unrivaled depths (>10 μm), and in addition, it has the unique ability to reveal diffusion properties inside the tissues. To locally probe the ECS dimension and diffusivity around synapses deep inside living brain tissues, the use of SWCNTs thus emerged as the tool of choice.

In order to identify synaptic areas into cultured hippocampal brain slices (Figure S1), we fluorescently labeled synapses using GFP-PSD95 lentivirus vectors (Figures 1 and S2). PSD95 is one of the most abundant proteins in excitatory synapses and is a common marker of postsynaptic areas. Synaptic imaging was performed in the CA1 region of the hippocampus at depth ranging from 10 to approximately 50 μm (Figure S3A) using a speckle based structured illumination technique known as HiLo microscopy.^16^ HiLo is a wide-field fluorescence microscopy method which provides excellent optical sectioning capabilities in thick biological samples, akin to confocal microscopy but at higher imaging rates and with simpler instrumentation. It requires the acquisition of two images: one using a uniform illumination and one with structured illumination (here the illumination transported through a multimode fiber is randomly structured by the speckle). These two images are used to extract the high and low frequency in-focus contents (from here, the acronym HiLo), leading to a full resolution in-focus image containing the entire frequency bandwidth of the imaging system (Figure 1C). The basic principle is that the in-focus high frequency content of the image can be extracted by high-pass filtering of the image acquired using uniform illumination, while the in-focus low frequency content can be obtained from contrast analysis of the image obtained with structured illumination.^17^ From the HiLo images, GFP positive clusters corresponding to synapses were then identified in living brain tissues (Figure 1C).

Biocompatible fluorescent SWCNTs were prepared by encapsulation with phospholipid–polyethylene glycol (PL–PEG) molecules. This coating minimizes nonspecific adsorption onto biological structures^18^ while preserving SWCNT luminescence brightness for single molecule experiments. Cultured hippocampal slices expressing GFP-PSD95 were incubated with PL–PEG coated SWCNTs, and slices were placed onto an NIR single molecule microscope (see Material and Methods). We focused on (6,5) SWCNTs emitting at 985 nm which are efficiently excited at 845 nm while minimizing light absorption by the tissue.^19^ Bright (6,5) SWCNTs were sparsely and individually detected at high signal-to-noise ratio with low autofluorescence (coming from biological structures or from out-of-focus nanotubes (Figure 1D), which constitutes a decisive asset to perform single molecule imaging at the required depth. Indeed, investigating relevant ECS structures inherently requires thick brain tissue preparations (generally a few hundred micrometers). Luminescent SWCNTs were imaged at 33 frames per second to grasp their rapid diffusion within the ECS (see Movie S1). Importantly, the SWCNT high aspect ratio and intrinsic rigidity play a decisive role here, slowing down nanotube diffusion in the ECS maze while ensuring high accessibility to nanoscale environments.^20^ These are unique features of these bright non-photobleaching 1D nanoparticles.

Superlocalization analysis of nanotube positions along their trajectories was performed as follows. For each recorded movie frame, we applied a 2D asymmetric Gaussian fitting analysis of the fluorescence profiles to decipher the nanotube centroids with subwavelength precision (∼50 nm) and nanotube length (long axis of the Gaussian fit). Taking into account the exciton diffusion range that decreases the apparent nanotube length in fluorescence images due to end quenching,^21^ the measured nanotube length distribution was centered around ∼600 nm (Figure S3B). This narrow distribution confirms the consistency and reproducibility of the nanotube preparation over 14 independent experiments (3 slices per experiment; 76 analyzed SWCNTs).

In order to explore the ECS environment around the synapses, the region around each GFP-PSD95 centroid was segmented by a series of concentric coronal areas of 100 nm widths (Figure 2A). A maximum distance of 1 μm from the synaptic centroids was considered, based on the average synapse density in hippocampal neurons (i.e., around 1 spine per μm of dendrite^22^). An image correlation analysis with SWCNT localizations allowed us to create a distance-to-synapse investigation of the ECS features. We first analyzed SWCNT local diffusivity in the different coronal areas. Local diffusivity was defined as the normalized local instantaneous diffusion along trajectories. For this, the two-dimensional mean-squared displacement (MSD) was calculated as a function of time intervals along a sliding window of 390 ms for each trajectory. By approximating the MSD as linear at short time intervals, a linear fit of the first 90 ms yielded *D*_inst_, the instantaneous diffusion coefficient. The normalized diffusivity was then obtained by calculating *D*_inst_/*D*_ref_ where *D*_ref_ is the calculated diffusion coefficient of carbon nanotubes freely diffusing in a fluid having the viscosity η_ref_ of the cerebrospinal fluid (CSF)^7^ (see Material and Methods).

**Figure 2:**
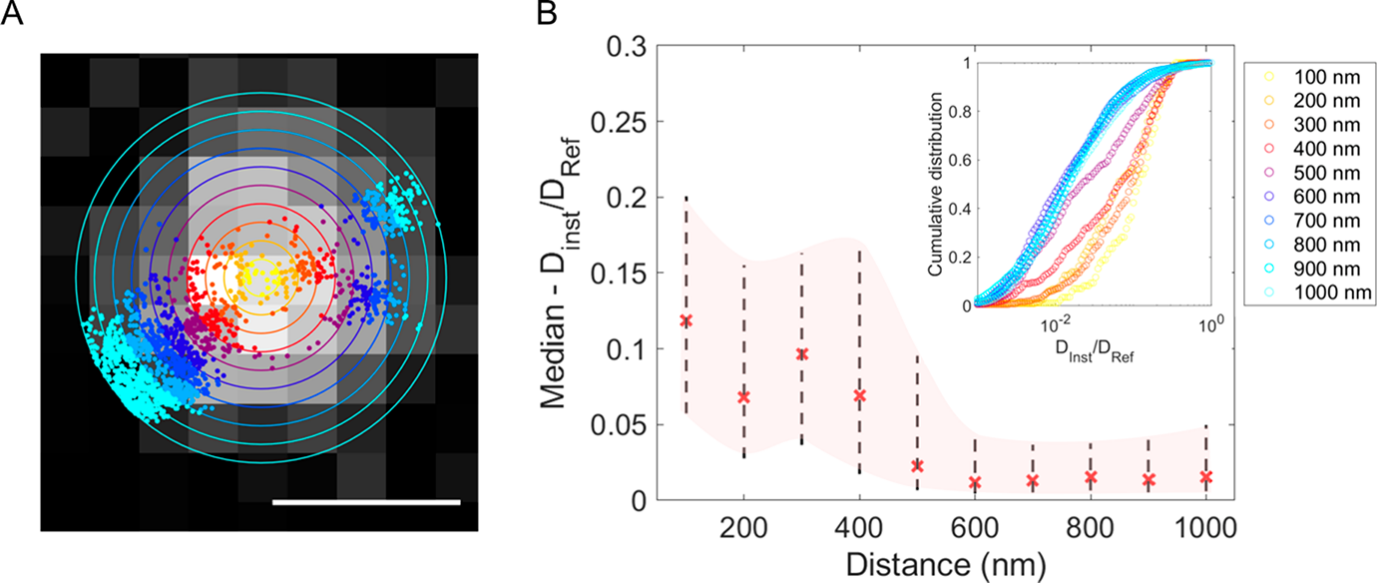
(A) Schematic of the analysis. Individual SWCNT trajectories were analyzed on coronal areas of 100 nm from the synaptic centroids. Scale bar is 1 μm. (B) Median (red crosses), 25th/75th percentile (shaded area), and cumulative distribution functions (inset) of the diffusivity values calculated at each coronal area for control conditions (31 SWCNTs). A sharp transition in the diffusivity values was observed between 400 and 500 nm from the PSD95 centroids.

Figure 2B shows median values and cumulative distributions (inset) of local diffusivities measured in different coronal areas around synapses. Clearly, a distance-to-synapse dependent behavior lies within submicron scales (Figure 2B). More specifically, a sharp transition in the diffusivity is observed between 400 and 500 nm revealing a specific diffusivity behavior around synapses where the particles undergo a 10-fold enhanced diffusivity as compared to farther away from synapses. In order to unambiguously assess the presence of this specific juxta-synaptic diffusion environment, a series of controls was performed. First, we simulated SWCNT trajectories assuming Brownian motion and randomly generated synaptic localizations to rule out that the generation of 100-nm-width coronal regions might bias apparent diffusivities into reduced coronal areas (see Material and Methods and Figure S4A,B. Furthermore, using the experimental SWCNT trajectories, we also generated “fake” (randomized) localizations of synapses in regions where no positive GFP-PSD95 signals were experimentally identified: no specific (enhanced) diffusivities were subsequently generated around the “fake” synaptic areas (Figure S4C). We thus conclude that a “juxta-synaptic” environment exists up to 500 nm away from synaptic centroids where the diffusivities are larger than in “non-juxta-synaptic” areas (areas between 500 and 1000 nm away from GFP-PSD95 centroids). We finally concluded that the presence of nanotubes in the juxta-synaptic regions did not alter synaptic spontaneous activity. For this, dissociated primary neuronal cultures were infected with GCaMP6, a calcium reporter, to monitor the spontaneous activity of excitatory synapses following exposure to SWCNTs (Figure S5A). For this control, we deliberately used a particle concentration resulting in imaged densities (∼4 × 10^10^ SWCNT.mL^–1^) far exceeding those observed in our brain slices to ensure that the vast majority of synapses were statistically exposed to a SWCNT. We report that the presence of SWCNTs, for tens of minutes, do not alter the frequency of synaptic transmission in active hippocampal neuronal network (Figure S5B).

Based on this partition of the ECS environment definition (juxta- or non-juxta-synaptic), we next ran an extensive characterization of the ECS features pooled into these regions (Figure 3). In addition to information on local diffusivity, the analysis of SWCNT localizations also provides information on the local ECS dimensions applying an analytical approach described previously.^13^ In short, SWCNT localizations were fitted to an ellipse over short periods of time (180 ms), where the shorter dimension represents the local ECS dimensions (ξ). Spatial maps of local diffusivities (*D*_inst_/*D*_ref_) and ECS local dimensions were thus generated (Figure 3A), revealing that SWCNT diffusion is heterogeneous in all ECS areas. Due to the high neuronal density of brain tissues, we cannot exclude that the non-juxta-synaptic region may include synapses from other dendrites of non-infected (nonlabeled) neurons, so that non-juxta-synaptic behavior might be contaminated by juxta-synaptic features. In addition, because this work does not superlocalize SWCNT in 3D (nor synaptic centroids), the depth of focus of our microscope does not discriminate juxta-synaptic areas along the *z* (optical) axis of the microscope, such that juxta-synaptic regions might also be contaminated by non-juxta-synaptic features. In any case, we found that the juxta-synaptic diffusivity is 6-fold faster than the non-juxta-synaptic region (median__juxta_ = 0.089; median__non-juxta_ = 0.014; *p* < 0.001; Figure 3B) which might in fact represent a lower fold due to the two possible “contaminations” just mentioned.

**Figure 3:**
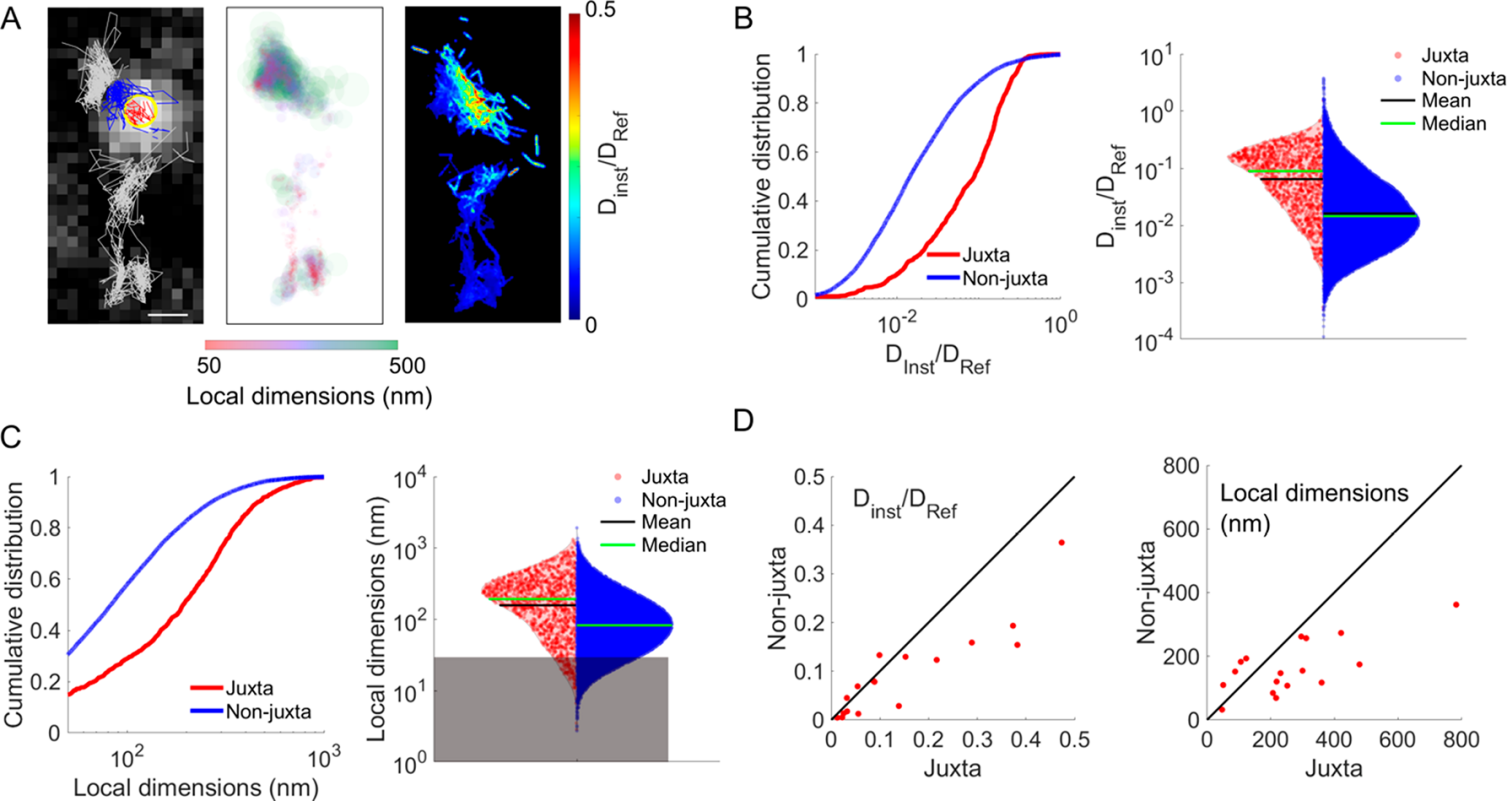
Analysis of untreated organotypic brain slices. (A) Examples of individual trajectories. Localizations were divided based on the relative position to a GFP-PSD95 centroid (left panel: juxta-synaptic in red, non-juxta-synaptic in blue). Localizations farther than 1 μm are depicted in gray. Superlocalization analysis gave further information on local ECS dimensions (central panel) and diffusivity (right panel). Scale bars are 2 μm. (B) Cumulative distributions and violin plot and of diffusivity and (C) local dimensions. Graphs show that the juxta-synaptic nanoenvironment bears specific local properties (*p* < 0.001). (D) Scatterplots comparing juxta- and non-juxta-synaptic diffusivity and local dimensions of individual GFP-PSD95 positive clusters, showing that the diffusivity is higher and the local dimensions larger in the juxta area even at the single-synapse level.

Figure 3C displays ECS dimension values in juxta- and non-juxta-synaptic domains. Similar to diffusivity, local ECS dimensions were highly heterogeneous, their widths ranging from around 50 nm (limited by the precision of our approach) to well above 1 μm. The vast majority (>70%) of local dimensions in the juxta-synaptic nanoenvironment were larger than 100 nm. Strikingly, the ECS local dimensions are significantly larger (∼2-fold) in the juxta-synaptic region as compared to the non-juxta-synaptic ones (median__juxta_ = 193 nm – median__non-juxta_ = 83 nm; *p* < 0.001; Figure 3C). Finally, we performed the comparison of diffusivity and local dimensions between juxta- and non-juxta-synaptic regions for each individual synaptic environment and represented each synapse on a scatter plot in Figure 3D. At the single synapse level, this analysis confirmed that higher diffusivity and larger local ECS dimensions are found in juxta-synaptic environments with respect to the local non-juxta-synaptic nanoenvironment. This observation has been confirmed by a matched paired analysis (*p* < 0.01).

In general, the broad shape of the cumulative distributions confirmed that the ECS is a highly heterogeneous milieu, where diverse local properties can impose a wide range of diffusivity and dimensional values. Indeed, in contrast to a free medium, diffusion in the brain ECS can be hindered by cell processes, astroglia, macromolecules of the matrix, wall drag, and the presence of charged molecules. In this environment, the diffusivity is also dependent on the hydrodynamic dimension of the diffusing probe, resulting in lower diffusivities for larger objects. Interestingly, median values of diffusivity and local dimensions evaluated at the level of individual juxta-synaptic regions are uncorrelated (Pearson’s *r* = 0.138; Figure S6A), suggesting that (*i*) the diffusivity of SWCNTs is mainly influenced by the molecular composition of the space, and (*ii*) spatial constrictions of cellular walls are not necessarily the central determinant of ECS diffusion inhomogeneities at the nanoscale near synapses.

As stated above, changes in neuronal activity are likely to alter ECS characteristics.^12,14,15^ We thus now question whether these changes also alter ECS diffusivity and morphology in the juxta-synaptic nanoenvironment. We used two classical protocols to either favor (bicuculline, BIC 40 μM) or decrease (tetrodotoxin, TTX 2 μM) neuronal activity (Figure 4A). As expected, incubation with BIC significantly increased the basal activity, whereas TTX suppressed it (Figure 4B). Additionally, no significant differences were detected on the dimension of the GFP-PSD95 clusters (*p* = 0.1231, Figure S7).

**Figure 4:**
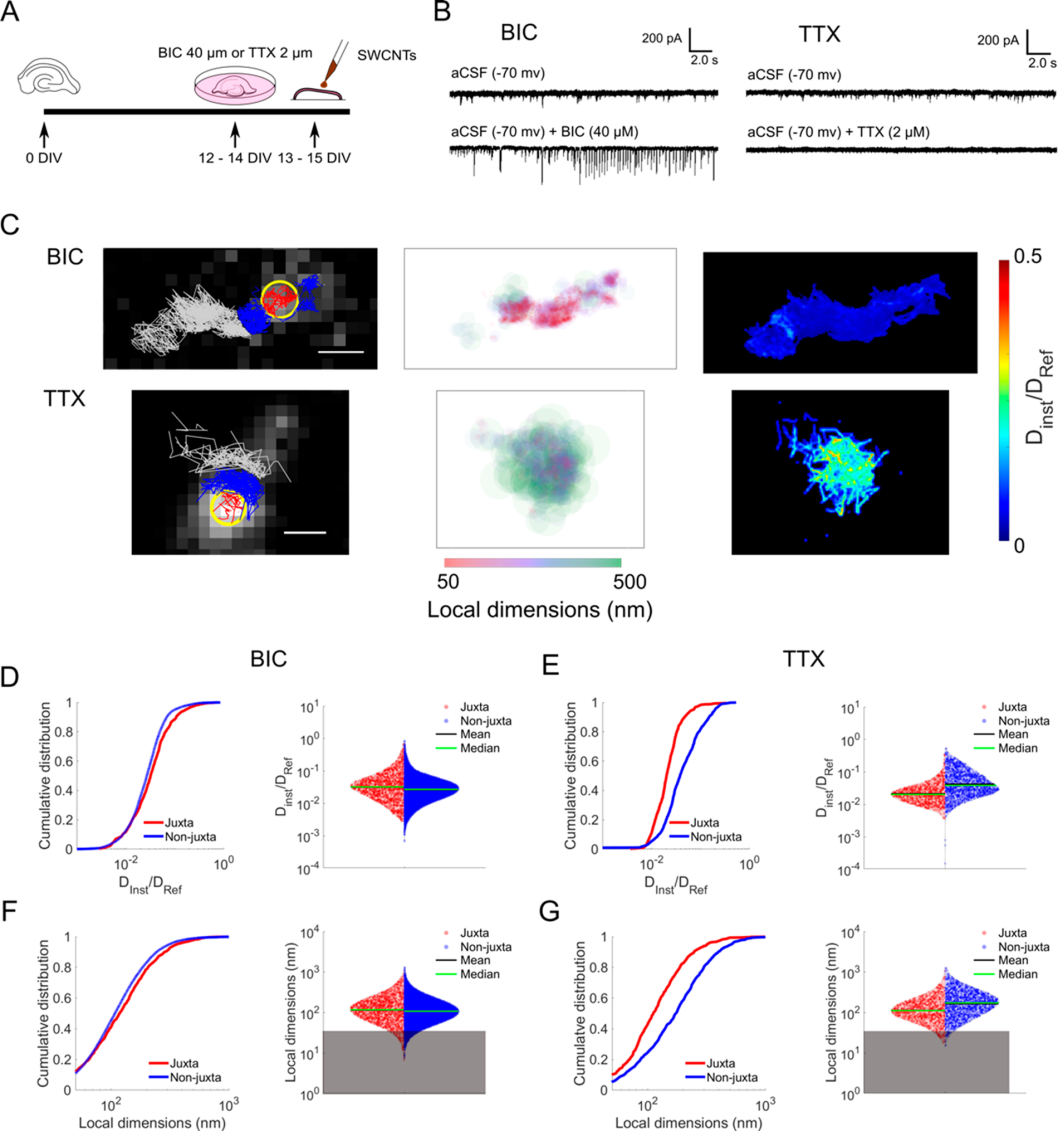
Stimulation of organotypic brain slices. (A) Schematic of the stimulation. BIC or TTX treatments were applied for 24 h prior to SWCNT incubation to respectively block the inhibitory action of GABA_A_ receptors or the sodium channels. (B) Representative electrophysiological traces of excitatory postsynaptic currents (EPSCs) from rat organotypic hippocampal slices DIV 12. Top traces recorded in the presence of aCSF only; bottom traces after addition of BIC or TTX in the medium to increase or decrease neuronal activity, respectively. (C) Examples of individual trajectories for BIC- and TTX-treated organotypic slices. As for control samples, localizations were divided based on the relative position to a GFP-PSD95 centroid (left panel: juxta-synaptic in red, extra-synaptic in blue). Localizations further than 1 μm are depicted in gray. Superlocalization analysis gave further information on local ECS dimensions (central panel) and diffusivity (right panel). Scale bars are 2 μm. (D) Cumulative distributions and violin plot of diffusivity for BIC-treated samples. Graphs show that BIC unified the synaptic microenvironment. (E) Cumulative distributions and violin plot of diffusivity for TTX-treated samples, showing a significant slowdown of diffusivity in the juxta-synaptic nanoenvironment. (F) Violin plot and cumulative distributions of local dimensions for BIC-treated samples, confirming the uniformity of the environment. (G) Violin plot and cumulative distributions of local dimensions for TTX-treated samples. For all graphs, the juxta-synaptic localizations are marked in red, while the non-juxta-synaptic ones are in blue. The gray boxes represent the localization precision of our analysis.

Figure 4C shows examples of trajectories of SWCNTs in hippocampal tissues exposed to BIC or TTX. Similar to untreated samples, we partitioned nanotube localizations based on their juxta- or non-juxta-synaptic position. We observed that modulation of neuronal activity is accompanied by changes of the ECS local environment around synapses. More precisely, comparing ECS domains in each condition, we found that for BIC-treated samples the difference in diffusivity between regions near or distant from the GFP-PSD95 centroid became less pronounced (median__juxta_BIC_ = 0.032, median__non-juxta_BIC_ = 0.027, Figure 4D), whereas TTX yielded significantly lower values of diffusivity in the juxta-synaptic environment compared to non-juxta-synaptic spaces (median__juxta_TTX_ = 0.020, median__non-juxta_TTX_ = 0.038, Figure 4E). A similar alteration/modification was detected after the analysis of local dimensions in the juxta- and non-juxta-synaptic regions (median__juxta_BIC_ = 118 nm, median__non-juxta_BIC_ = 108 nm, Figure 4F; median__juxta_TTX_ = 112 nm, median__non-juxta_TTX_ = 174 nm, Figure 4G).

We next focus on the juxta-synaptic region to compare the different conditions (Figure 5). SWCNT diffusivity was slowed and ECS local dimensions were shrank in both BIC and TTX treatments when compared to control conditions (Figure 5A,B, *p* < 0.001). This observation suggests that the neuronal network can accommodate to neuronal activity changes (either increase or blockade) through ECS regulation. Finally, Figure 5 also indicates that in the juxta-synaptic region distributions of local diffusivity and dimensions of treated samples are less disperse than in control conditions, suggesting that BIC and TTX treatments standardized the juxta-synaptic environment.

**Figure 5:**
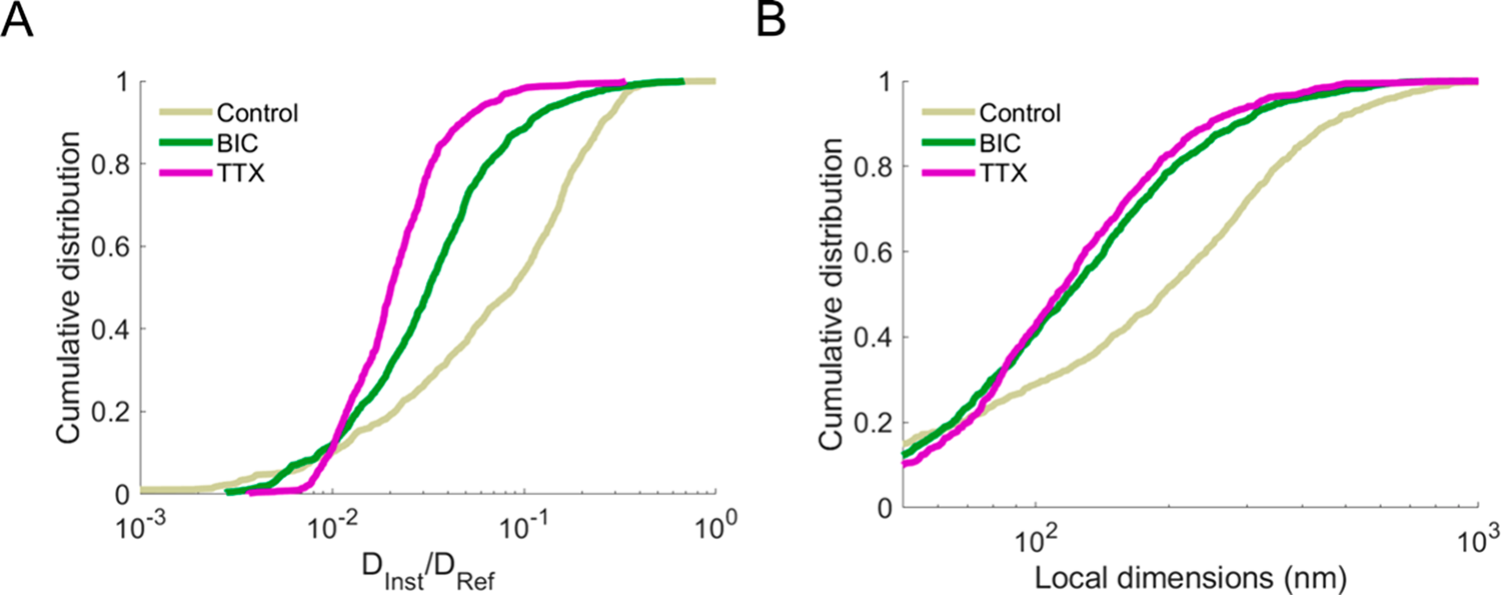
Comparison of cumulative distributions of (A) local diffusivity and (B) local dimensions for control (gray), BIC-treated (green), and TTX-treated (purple) organotypic slices. When compared to control samples, both BIC and TTX slowed the SWCNT diffusivity in the juxta-synaptic nanoenvironment and narrowed down the local dimensions (*p* < 0.001). Local diffusivity in TTX-treated samples was significantly slower than in BIC-incubated tissues (*p* < 0.001), but the local dimensions remained comparable between the two conditions.

A significant and global decrease in the ECS volume fraction and an increase in diffusion barriers have been reported during neuronal activity and pathological states.^23^ These changes were related to cell swelling, cell loss, astrogliosis, rearrangement of neuronal and astrocytic processes, and changes in the extracellular matrix. Plastic changes in ECS volume, tortuosity, and anisotropy can also affect the communicaton between neurons and other cell types (e.g., astrocytes, oligodendrocytes).^5,6,24^ Here, TTX-treated samples have slower diffusivity than the ones incubated with BIC (*p* < 0.001), but the local dimensions remain comparable between conditions, suggesting that the differences between BIC and TTX relies on the chemical modifications of the juxta-synaptic region. This is further supported by the correlation analysis evaluated for individual GFP-PSD95 positive clusters, which revealed a higher correlation between local diffusivity and dimensions in the juxta-synaptic region for BIC-treated with respect to TTX-treated samples (Figure S6B,C).

Altogether, our study revealed the existence of a juxta-synaptic ECS nanoenvironment within 500 nm from excitatory synapses in hippocampal brain slices, not accessible with previous approaches due to limited resolution. This observation was possible by correlating the dynamics and superlocalization of NIR-emitting carbon nanotube with HiLo microscopy of labeled synapses in live brain slices. Increasing or decreasing synaptic activity specifically modified the ECS diffusion and morphological parameters in the juxta-synaptic region. Such regulation of the ECS nanoenvironment around synapses would strongly influence the diffusion of neurotransmitters and modulators in the brain tissue, impacting neuronal network physiology and pathology.

## Supporting Information Available

Movie of raw acquisition of CNTs (AVI)
Materials and Methods, including figures (PDF)

## Supplementary Material

nl1c04259_si_001.avi
