# Supplementary material for: Near-Infrared Carbon Nanotube Tracking Reveals the Nanoscale Extracellular Space around Synapses
Source: Nano Lett. 2022 Aug 29;22(17):6849–56. doi: 10.1021/acs.nanolett.1c04259 (PMC9479209; doi:10.1021/acs.nanolett.1c04259)
Supplement: Supplementary file 2 — nl1c04259_si_002.pdf [file nl1c04259_si_002.pdf]

Supporting information for

Near-Infrared Carbon Nanotube Tracking Reveals the Nanoscale Extracellular Space around Synapses

Chiara Paviolo[†,#], Joana S. Ferreira[‡,#], Antony Lee[†], Daniel Hunter[‡], Ivo Calaresu[‡], Somen Nandi[†], Laurent Groc[‡,*], Laurent Cognet[†,*]

[†]*Université de Bordeaux, Institut d'Optique & Centre National de la Recherche Scientifique, UMR 5298, 33400 Talence, France*

[‡]*Université de Bordeaux, Interdisciplinary Institute for Neuroscience, UMR 5297, 33076 Bordeaux, France*

[#] equal contribution

* Email: laurent.groc@u-bordeaux.fr

* Email: laurent.cognet@u-bordeaux.fr

MATERIALS AND METHODS

Rat organotypic slice preparation

Organotypic slice cultures were prepared as previously described in Paviolo *et al.*[1]. Hippocampal slices (350 µm) were obtained from postnatal day 5 to 7 Sprague-Dawley (Janvier Labs) rats using a McIlwain tissue chopper and then placed in dissection medium containing (in mM): 175 sucrose, 25 D-glucose, 50 NaCl, 0.5 $CaCl_2$, 2.5 KCl, 0.66 KH_2PO_4, 2 $MgCl_2$, 0.28 $MgSO_4$-7H_2O, 0.85 Na_2HPO_4-12H_2O, 2.7 $NaHCO_3$, 0.4 HEPES, 2×10–5% phenol red, pH 7.3. Slices were kept at 4°C until they were transferred to hydrophilic polytetrafluoroethylene (FHLC) membranes (Millipore) set on Millicell Cell Culture Inserts (Millipore), containing pre-warmed culture medium (50% Basal Medium Eagle, 25% Hank's balanced salt solution, 25% horse serum, 0.45% D-glucose, and 1mM L-glutamine) and cultured for up to 14 days at 35°C / 5% CO_2 and the medium replaced every 2 to 3 days.

Lentivirus construction and slices infection

Lentivirus transfer vector construct (FHUG+W) expressing PSD95 tagged with GFP[2], was produced at the Vectorology Platform (INSERM US 005 – CNRS 3427 – TBMCore, Université de Bordeaux, France) by transfection with a 3 viral vector system — mock with the PSD95-GFP insert, pCMV-Δ8-9 (encapsulation plasmid), and VSV-G (cDNA encoding the envelope glycoprotein of vesicular stomatitis virus) — in FT-HEK293 cells. Lentivirus supernatants were concentrated by centrifugation concentration filter (Centricon) and a final titer of 2.14^8 virus particles/ml was obtained. 1 µl of the concentrated lentivirus was added to FHLC membranes, cut to the slice size, and immediately inverted over the slice at 4 days of culture *in vitro* (DIV 4) for 24h. After FHLC membrane removal, medium was replaced with fresh one.

Confocal images of infected slices

Images of GFP-PSD95 expressing neurons in organotypic slices at DIV 12 were taken in an inverted Leica DMI 6000 microscope (Leica Microsystems, Wetzlar, Germany) equipped with a confocal head Yokogawa CSU-X1 (Yokogawa Electric Corporation, Tokyo, Japan), a sensitive Quantem camera (Photometrics, Tucson, USA), using a HCX PL APO CS 63× oil 1.32 NA objective and a 491 nm diode laser. This system was controlled by MetaMorph software (Molecular Devices, Sunnyvale, USA).

SWCNT preparation

SWCNTs were prepared as previously described with minor modifications[1]. Briefly, 1 mg of HiPco synthesized carbon nanotubes (from Rice University) was suspended with 50 mg of monofunctional phospholipid-polyethylene glycol (PL-PEG) (#mPEG-DSPE-5000, Laysan Bio) in 10 ml of deuterium

oxide (Sigma Aldrich). To individually disperse the nanotubes, a 15 min homogenization at 19,000 rpm followed by an 8 min tip sonication at 20W were applied to the solution. SWCNT bundles and impurities were further precipitated by centrifugation at 3,000 rpm for 60 min. 70–80% of the supernatant was then collected and stored at 4 °C.

Slice stimulation and SWCNT incubation

For GFP-PSD95-infected slice stimulation, 40 µM of (-)-Bicuculline methochloride (BIC, TOCRIS) or 2 µM of tetrodotoxin (TTX, TOCRIS) were applied for 24 hours to respectively block the inhibitory action of $GABA_A$ receptors or the sodium channels at DIV 12-14. After the incubation time, slices returned in fresh culture medium. SWCNTs were incubated in the cultures 2 hours prior imaging. 3 µl of SWCNT solution was mixed with 100 µl of culture medium and incubated with the GFP-PSD95-infected slices at 35 °C / 5% CO_2. Slices were imaged for up to 1 h in HEPES-based artificial cerebrospinal fluid (HEPES-aCSF) containing (in mM): 130 NaCl, 2.5 KCl, 2.2 $CaCl_2$, 1.5 $MgCl_2$, 10 HEPES, and 10 D-glucose.

Electrophysiology of BIC and TTX treated slices

For electrophysiological experiments, whole-cell voltage-clamp recordings were taken from CA1 pyramidal cells in organotypic hippocampal slice cultures at DIV 12. Slices were transferred to the recording chamber, perfused with 32°C artificial cerebrospinal fluid (aCSF) composed of (in mM): 126 NaCl, 3.5 KCl, 2 CaCl2, 1.3 MgCl2, 1.2 NaH2PO4, 25 NaHCO3 and 12.1 glucose. For pharmacological manipulations, aCSF perfusion was supplemented with bicuculline (BIC, 40µM) to block GABAR-mediated inhibition; or with tetrodotoxin (TTX, 2µM) to block action potential-driven synaptic communication. Recording pipettes with a 5-6 MΩ resistance were filled with intracellular solution (in mM): 134 caesium methanesulfonate, 10 HEPES, 0.5 EGTA, 4 Mg-ATP, 0.3 Na-GTP and 4 NaCl. After achieving whole-cell configuration, cells were voltage-clamped at -70mV to record excitatory postsynaptic currents, using a MultiClamp™ 700B amplifier (Molecular Devices), and digitised by Axon Digidata 1550B (Molecular Devices). Baseline recordings were first obtained in the absence of BIC or TTX. Current traces were prepared using Clampfit (v10.7) software.

Correlative imaging of SWCNT and GFP-PSD95

Imaging was performed on a customized epifluorescent microscope (Nikon) equipped with a water-cooled EM-CCD camera (ProEM-HS, Princeton Instrument). A standard 4× objective (NA 0.1, Nikon) was initially used to check the CA1 position in the hippocampal slice using a white light fibre positioned with an angle of ~ 30° above the sample. A 845 nm laser was used to excite individual (6,5) SWCNTs at their phonon sideband (λ_{exc} = 845 nm / λ_{em} = 986 nm) with a circular polarized excitation. Images

were collected using a water immersion 60× objective (NA 1.0, Nikon) using an exposure time of 30 ms (33 frames per second). The fluorescence of GFP-PSD95 was excited using a 488 nm laser (Coherent) and recorded by a HiLo modality [3]. For HiLo, switching between uniform or structured wide-field illumination was performed using a Sparq module (Bliq Photonics) based on the use of a multimode fiber. Visible images were collected at the end of each SWCNT recording. Recording depths were measured as the distance between the brain slice surface identified under oblique white light illumination and the point of recording using the motorized focusing meter of the microscope.

Analysis of GFP-PSD95 cluster areas

GFP-PSD95 positive clusters selected for analysis were subjected to an intensity threshold to define the cluster, followed by the measurement of the selected area with ImageJ version 1.53j.

Super-localization analysis

Super-localization of the SWCNT centroids was obtained by fitting the NIR images with two-dimensional asymmetric Gaussian functions having arbitrary orientations. Three consecutive images were averaged for each fit to improve the localization precision (~50 nm in water). Eventual drifts were corrected using an immobile SWCNT in the field of view. SWCNT coordinates were then interconnected to reconstruct nanotube trajectories. GFP-PSD95 centroids were also estimated by fitting the visible images with two-dimensional asymmetric Gaussian functions. The distance between the SWCNT and the GFP-PSD95 centroids gave the relative position of the nanotube to a synapse. Diffusivity and local dimension analysis were computed on individual trajectories in coronal areas of 100 nm from the synaptic centroids.

Local ECS dimensions were estimated as previously described. Briefly, SWCNT localizations (typically 5000 points) were fitted to an ellipse for time windows of 180 ms. The shorter dimension of the ellipse was then used to define the local ECS dimensions (ξ) as described in [1].

Local relative diffusivity

Analysis of individual diffusing SWCNTs in the ECS of live tissues was performed as follows. For each trajectory, the SWCNT length was estimated using the distribution of the longest axis of the 2D asymmetric Gaussian fits for negligible SWCNT movements (displacements between consecutive images < 40 nm) corrected by the point-spread function of the microscope and the exciton diffusion length[4].

The instantaneous mean square displacement (*MSD*) was then calculated for each trajectory as a function of time intervals Δt using sliding windows of 390 ms. For short time delays (90 ms), the two-dimensional MSD can be approximated by a linear slope,

$$MSD(t) = 4D_{inst}\Delta t \qquad (1)$$

therefore allowing the definition of D_{inst}, the instantaneous diffusion coefficient. The localisation precision was estimated from the intercept of the MSD axis at t=0 (~ 50 nm). The local relative diffusivity was defined as the ratio between D_{inst} and the value of free diffusion (D_{ref}) that the considered SWCNT would have in a fluid bearing the viscosity of the cerebrospinal fluid (η_{ref}):

$$D_{ref} = \frac{3k_B T ln(2\varphi)}{8\pi\eta_{ref}L} \qquad (2)$$

where k_B is the Boltzmann constant, T is the temperature, φ is the SWCNT aspect ratio and L is the nanotube length. For visualization purposes, the spatial diffusivity maps were convoluted with a 2D Gaussian of 50 nm full width at half maxima.

Simulations of Brownian motion

To validate the analysis of local relative diffusivity in 100 nm width coronal regions, the same analysis described in the previous paragraph was applied on simulated trajectories. For this, n = 30 trajectory of diffusing SWCNTs were simulated by computing the cumulative sums of 5000 normally distributed random displacements per trajectory (dx ~ Normal $(0, \sigma^2)$, dy ~ Normal $(0, \sigma^2)$, where σ=1). Positions of the synaptic centers were randomly generated along the trajectory. MSD and local relative diffusivity values within different coronal areas were calculated as previously described for experimental trajectories. For display, the distributions of local relative diffusivity were scaled according to Eq. 1 (using D_{inst}= 5.38 $\mu m^2/s$ with an acquisition time of 30 ms typical of experimental data)) and Eq. 2 (assuming standard laboratory conditions of T of 35 °C, $\eta_{ref} = 10^{-3}$ Pa.s, SWCNT typical length of 500 nm and diameter of 5 nm for calculating D_{ref}).

Statistics

A total of 76 SWCNTs has been analysed in this study (31 for control experiments, n = 6; 31 for BIC-treated samples, n = 6; 14 for TTX experiments, n = 4). Image analysis was performed using custom MATLAB (MathWorks) and Python scripts unless specified otherwise. Statistical analyses were performed in GraphPad Prism 6.01 software or MATLAB. Empirical cumulative distributions were analysed by Kolmogorov–Smirnov (KS) test. Comparison between juxta- and non-juxta-synaptic diffusivity of individual GFP-PSD95 positive clusters was analysed by Wilcoxon matched-pairs signed rank test, while local dimensions were analysed by Paired t-test. GFP-PSD95 area comparison was analysed by 1-way ANOVA. Statistics for linear correlation was evaluated with Pearson's r, using a Student's t distribution for the transformation of the correlation. For all statistical tests, the level of significance was set to $p < 0.05$. Discrete data are represented as mean ± standard error of the mean (SEM).

Neuronal Primary Cultures

All procedures were approved by the local ethic authorities and performed in accordance with the UE guidelines (2007/526/CE and 2010/63/UE). Pure cultures of dissociated hippocampal neurons were prepared as previously reported.[5,6] Briefly, hippocampi were isolated from Sprague-Dawley rat embryos (E18) to obtain neuronal and glial cells. Poly-lysine-coated Petri dishes (Ø 60 mm, Corning®) were used to plate (~ 10 cells/mm^2) and grow a glial cells carpet in a high-serum culture medium (Minimum Essential Medium 1×, 10% Horse Serum, 1% GlutaMAX, 0.35% D-Glucose, 1% Sodium pyruvate; Gibco™) for 15 days in vitro (DIV). This was essential to subsequently provide metabolic support for hippocampal neurons. These latter were plated (~ 245 cells/mm^2) onto poly-lysine-coated glass coverslips, which were in turn suspended at the interface with 2 weeks-old pure glial culture (1 Petri dish hosted 4 coverslips). Since then, serum-free culture medium (Neurobasal-Plus, 2% B27-Plus, 0.5 mM GlutaMAX; Gibco™) supplemented with cytosine β-D-arabinofuranoside hydrochloride (Ara-C, 2 μM final; Sigma-Aldrich®) was used to foster neuronal cultures growth, while limiting glial proliferation. Co-cultures were kept at 37 °C, 5% CO_2 up to 21 DIV. Once a week half of medium was replaced with Ara-C-deprived fresh one.

Neuronal Cultures Transfection

Upon ex-vivo reorganization of synaptic networks, primary cultured neurons display temporally structured and spontaneous electrical activity. To explore synaptic activity upon acute SWCNTs exposure, neuronal cells were transfected by $Ca_3(PO_4)_2$ method with a GCaMP6f construct (Plasmid #52924, Addgene), as fluorescent reporter for calcium dynamics.[7] In particular, 0.5 μg DNA of transgene per glass coverslip were precipitated in a tube and added drop-wise on top of neuronal cultures at 8 DIV. Transfected cultures were allowed to express the exogenous gene for at least one week, after which neuronal activity was studied.

Calcium Live Imaging (see Fig. S5)

Synaptic calcium dynamics were studied on hippocampal neurons grown for 15-21 DIV. Cultures were equilibrated for 5 minutes at room temperature (RT) in extracellular saline solution of composition (mM): 130 NaCl, 2.5 KCl, 2.2 $CaCl_2$, 1.5 $MgCl_2$, 10 HEPES, and 10 glucose (pH adjusted to 7.4 with NaOH; osmolarity ~ 300 mOsm). Samples were placed in a recording chamber mounted on an upright microscope (Eclipse Ni-E, Nikon) equipped with a confocal scanner unit (CSU-X1, Yokogawa). Genetically encoded Ca^{2+} reporter was excited at 488 nm with a 200mW laser diode (L4Cc, Oxxius) at 4% power. Excitation light was separated from the emitted using a quad band polychroic mirror

(405/488/561-568/635-647, Chroma®); emitted light was further passed on a 525/50 nm single-band bandpass filter (BrightLine®). Neurons were imaged with a 60× water dipping objective (CFI Apo NIR, 1 N.A.) and frames were continuously acquired each 150 ms using an Evolve® 512 EMCCD camera (Teledyne Photometrics). The imaging system was controlled by an integrated acquisition software (NIS-Elements AR, Nikon). Extracellular saline was renewed prior recording, which was conducted at RT. Spontaneous neuronal activity was monitored for 8-10 minutes before and after SWCNTs (35 ng/mL corresponding to ~40.10^9 SWCNT per mL considering 500 nm nanotube length typical) incubation (5 minutes). On average 7 regions of interest (ROI) per neuron were drawn around active spines (Fig. S5A, left snapshot). Synaptic events were count in each ROI to obtain median interevent intervals (IEIs) and frequency values of spines. The activity of the latter was thus used to provide an estimate of single neurons behaviour after nanoparticles exposure. From 5 different culture series, a total of 17 neurons from 17 different glass coverslips were studied. Images were analyzed with ImageJ software (NIH), and the corresponding time series were studied with Clampfit software (pClamp suite, 11 version; Axon Instruments) in off-line mode. Intracellular Ca^{2+} transients were expressed as fractional amplitude increase ($\Delta F/F_0$, where F_0 is the baseline fluorescence level and ΔF is the rise over baseline). Statistical analysis was performed using Prism 9 software (GraphPad): distribution normality was addressed with D'Agostino and Pearson omnibus normality test. Non-parametric tests were used in both non-normally distributed data and unequal variances among tested conditions. Accordingly, statistics between the two dependent variables were performed either with paired t-test or Wilcoxon signed-ranks test.

TABLES

Table 1

	**Control**	**BIC**	**TTX**
**# SWCNTs**	31 (n = 6)	31 (n = 4)	14 (n = 4)
**# GFP-PSD95s**	17	18	9

Summary of the experimental repetitions. The number of SWCNTs denote the number of trajectories analysed, while *n* corresponds to the number of different brain slices imaged. The numbers of GFP-PSD95 positive clusters represent the quantity of the juxta-synaptic nanoenvironments, meaning that SWCNTs not always reached the proximity of a PSD cluster Qualitatively, in TTX-treated samples, SWCNT were more constrained.

FIGURES

Figure S1

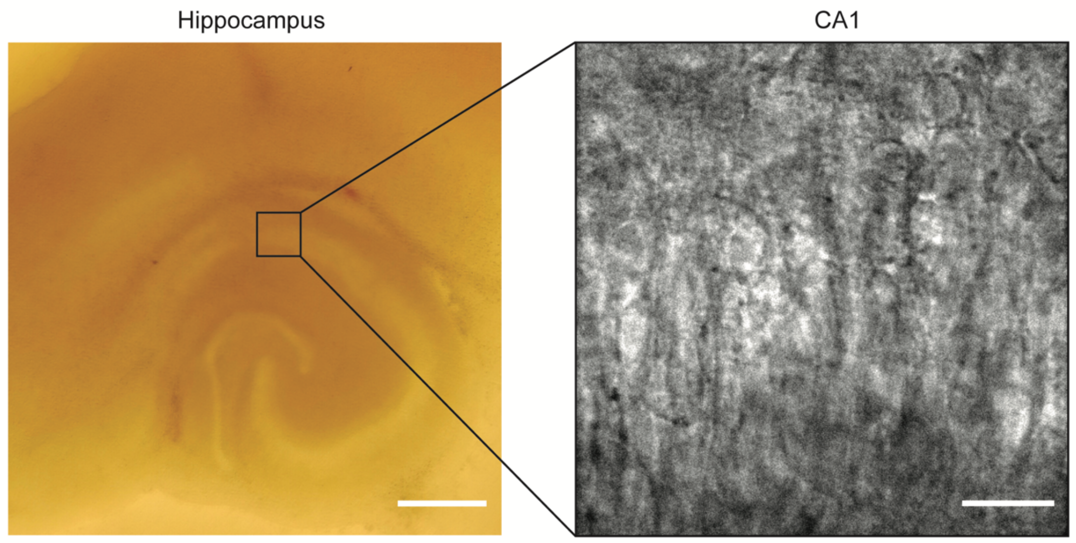

Bright field images of organotypic brain slices of whole hippocampus (left: low magnification obtained with a stereoscope) and of the CA1 stratum pyramidale (high magnifition 60x oil obtained with a widefield microscope. Scale bars are 300 µm in the whole slice (left) and 25 µm for the CA1 field (right).

Figure S2

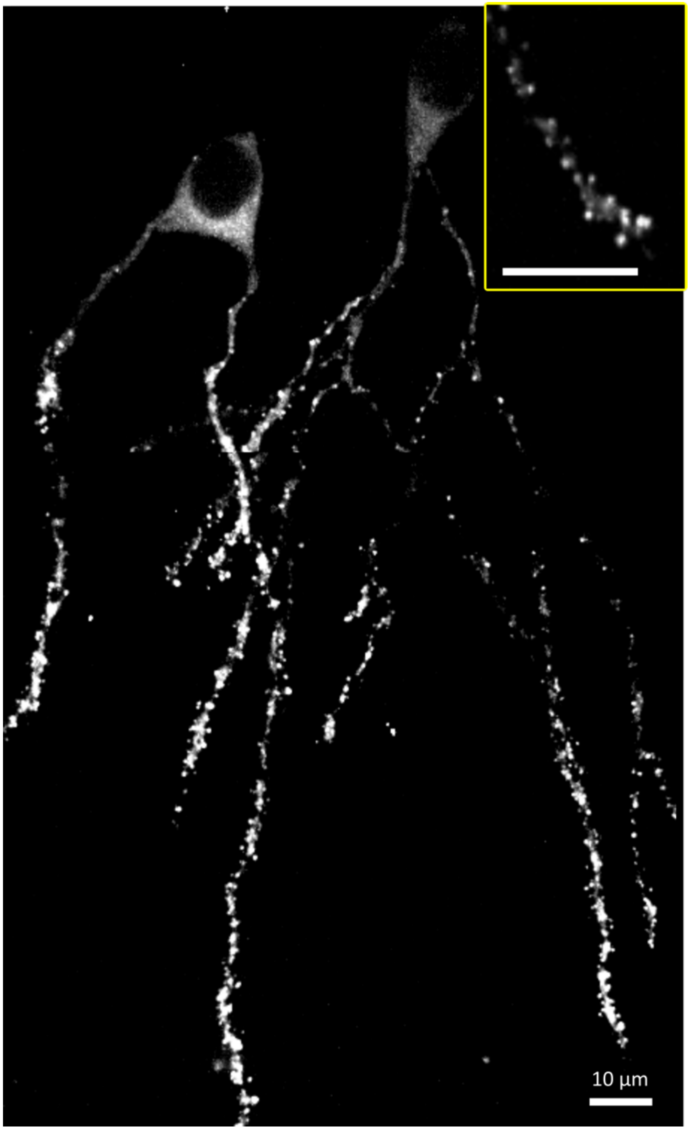

Image of GFP-PSD95 positive neurons with individual synapses (inset). Scale bars are 10 µm.

Figure S3

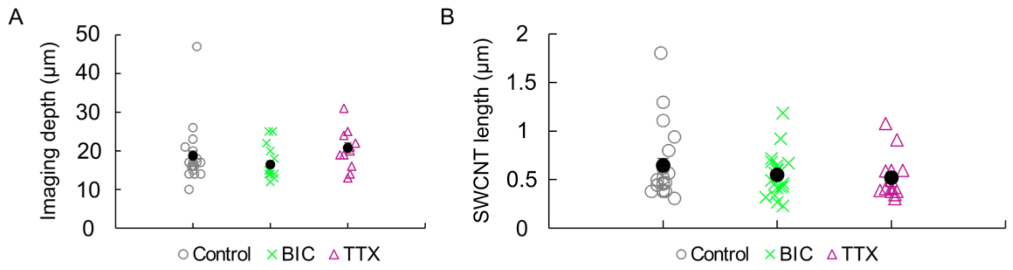

Summary of the experimental conditions in terms of imaging depth (A) and SWCNT length (B) in control, BIC, and TTX conditions.

Figure S4

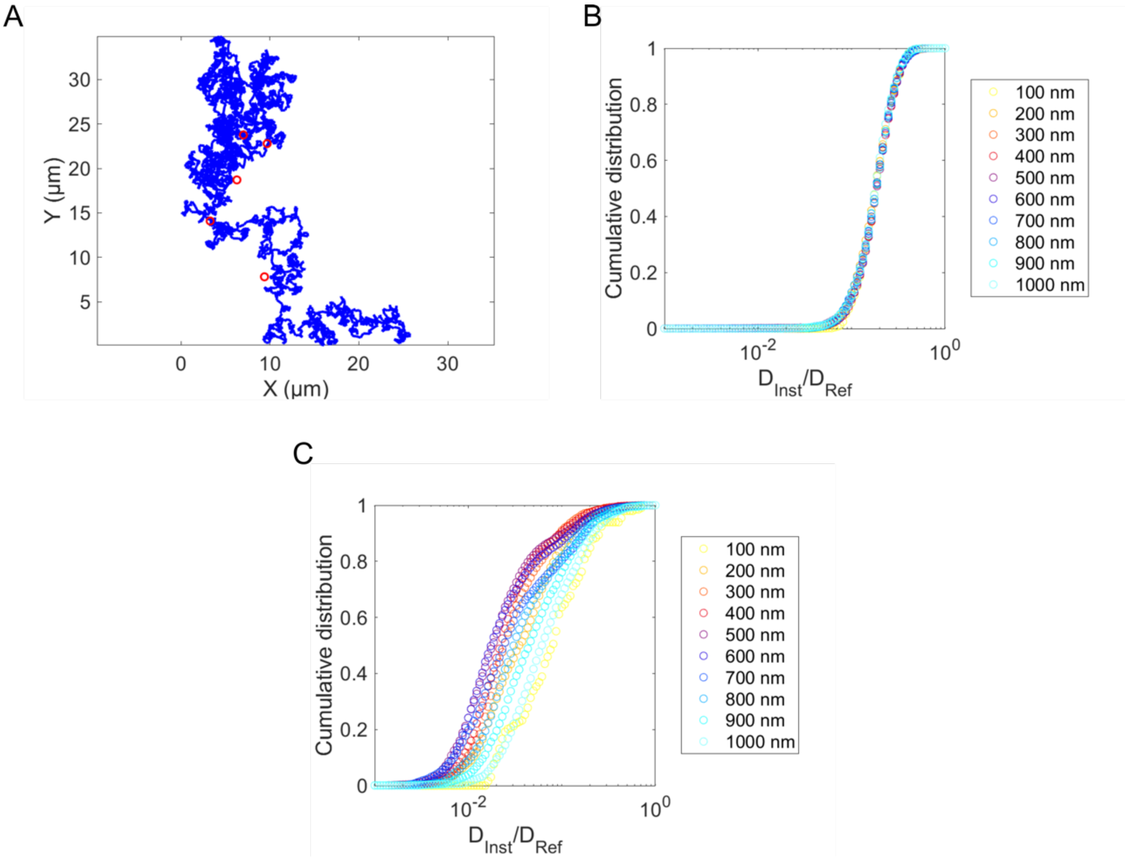

Negative controls for coronal areas. A) Simulated trajectories based on Brownian motion and randomly generated synaptic localization (in red, radius = 400 nm). B) Cumulative distribution functions of diffusivity in each coronal area for the simulated trajectories (N = 30) as in (A). C) From experimental trajectories, cumulative distribution functions of diffusivity in coronal area defined around “fake” synapses randomly generated in regions where no GFP positive clusters were experimentally identified. No monotonous distance-to-synapse dependency is found.

Figure S5

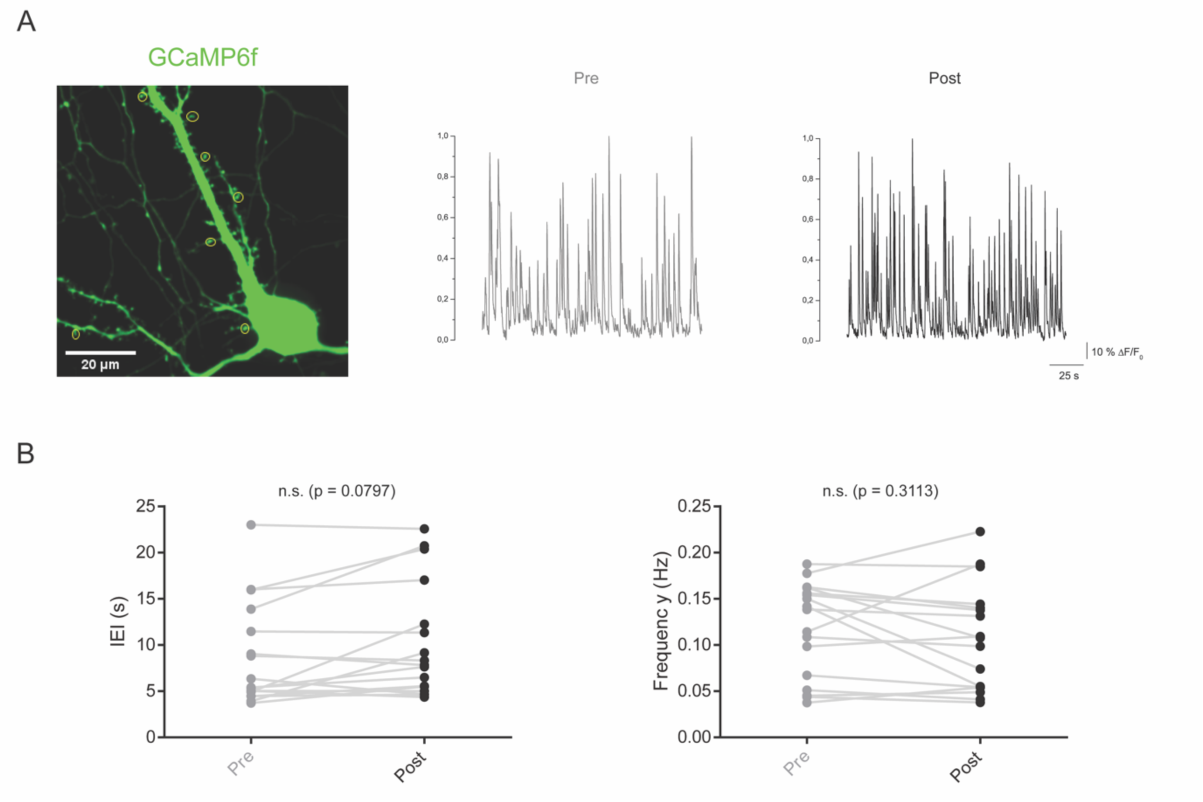

Neuronal calcium dynamics recording from spines upon acute SWCNTs exposure. A) On the upper left, a snapshot of a representative field with a hippocampal neuron is provided together with circular ROIs (yellow) drawn around spines from which activity was monitored. On the right side, representative fragments of recorded traces for each condition are shown. Calcium transients are expressed as fractional amplitude increase. B) Before and after (SWCNT administration) plots show averaged values of spines activity for each recorded neuron. Slight rising or dropping of interevent intervals and frequency values could be sometimes observed as expected by random chance. As denoted by *p-values* in such experimental conditions no significative differences have been detected *pre* and *post* SWCNTs administration.

Figure S6

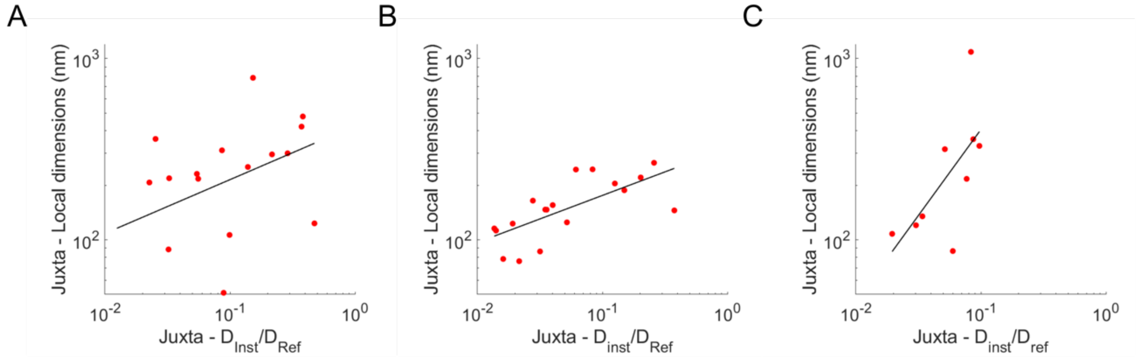

Correlation of local dimensions and diffusivity in the juxta-synaptic nanoenvironment on individual GFP-PSD95 clusters for control (A), BIC (B), and TTX (C) conditions. Median values of the parameters only showed a low or mild correlation for control and TTX samples (Pearson's r 0.375 and 0.533, respectively), suggesting that the diffusivity of SWCNTs in these environments was mainly influenced by the molecular composition of the space. Analysis of BIC-treated samples revealed a higher correlation (Pearson's r 0.656) between local dimensions and diffusivity.

Figure S7

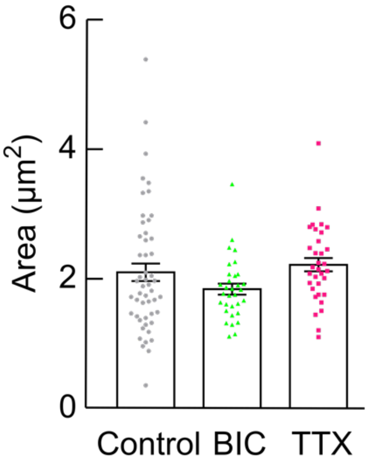

Comparison between PSD95 cluster areas in control, BIC, and TTX conditions. Treatment did not significantly change the size of PSD95 positive clusters, used for the corona definition.

REFERENCES

(1) Paviolo, C.; Soria, F. N.; Ferreira, J. S.; Lee, A.; Groc, L.; Bezard, E.; Cognet, L. Nanoscale Exploration of the Extracellular Space in the Live Brain by Combining Single Carbon Nanotube Tracking and Super-Resolution Imaging Analysis. *Methods* **2020**, *174*, 91–99.

(2) Porras, G.; Berthet, A.; Dehay, B.; Li, Q.; Ladepeche, L.; Normand, E.; Dovero, S.; Martinez, A.; Doudnikoff, E.; Martin-Négrier, M.-L.; Chuan, Q.; Bloch, B.; Choquet, D.; Boué-Grabot, E.; Groc, L.; Bezard, E. PSD-95 Expression Controls L-DOPA Dyskinesia through Dopamine D1 Receptor Trafficking. *J. Clin. Invest.* **2012**, *122* (11), 3977–3989.

(3) Lim, D.; Ford, T. N.; Chu, K. K.; Mertz, J. Optically Sectioned in Vivo Imaging with Speckle Illumination HiLo Microscopy. *J. Biomed. Opt.* **2011**, *16* (1), 016014-016014–016018.

(4) Oudjedi, L.; Parra-Vasquez, A. N. G.; Godin, A. G.; Cognet, L.; Lounis, B. Metrological Investigation of the (6,5) Carbon Nanotube Absorption Cross Section. *J. Phys. Chem. Lett.* **2013**, *4* (9), 1460–1464.

(5) Kaech, S.; Banker, G. Culturing Hippocampal Neurons. *Nat. Protoc.* **2006**, 1 (5), 2406–2415.

(6) Johansson, E. M.; Bouchet, D.; Tamouza, R.; Ellul, P.; Morr, A.; Avignone, E.; Germi, R.; Leboyer, M.; Perron, H.; Groc, L. Human Endogenous Retroviral Protein Triggers Deficit in Glutamate Synapse Maturation and Behaviors Associated with Psychosis. *Sci. Adv*. **2020**, 6 (29), 708–725.

(7) Chen, T.; Wardill, T. J.; Sun, Y.; Pulver, S. R.; Renninger, S. L.; Baohan, A.; Schreiter, E. R.; Kerr, R. A.; Orger, M. B.; Jayaraman, V.; Looger, L. L.; Svoboda, K.; Kim, D. S. Ultrasensitive Fluorescent Proteins for Imaging Neuronal Activity. *Nature* **2013**, 499 (7458), 295–300.
